# Supplementary material for: Interfacial and Foaming Properties of Tailor-Made Glycolipids—Influence of the Hydrophilic Head Group and Functional Groups in the Hydrophobic Tail
Source: Molecules. 2020 Aug 20;25(17):3797. doi: 10.3390/molecules25173797 (PMC7504461; doi:10.3390/molecules25173797)
Supplement: Supplementary file 1 [file molecules-25-03797-s001.pdf]

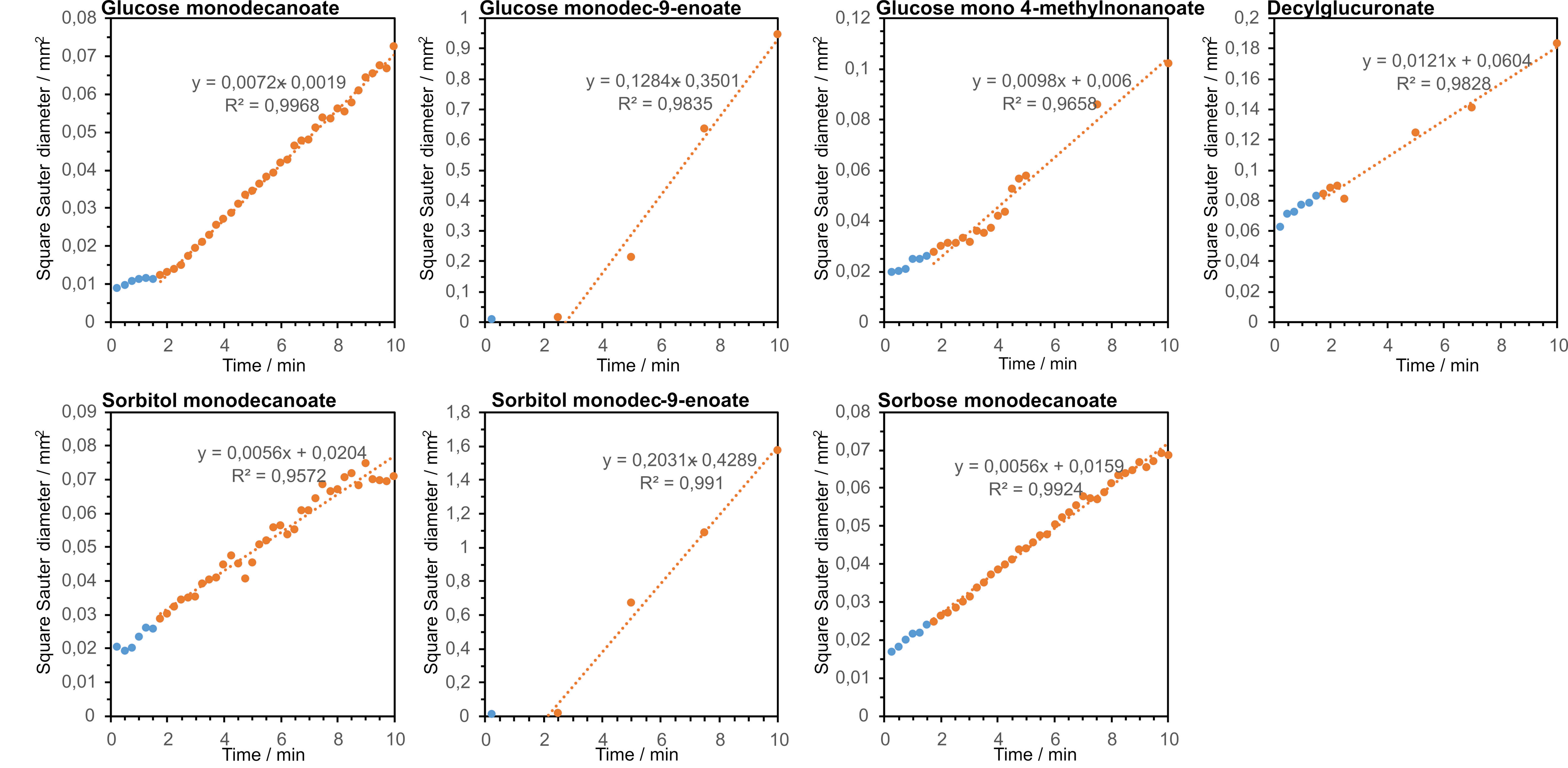

Supplementary Figure S1. Square Sauter diameter versus time plots for foams stabilized by the glycolipids.
